# Comprehensive review of biosynthesis, plant function, metabolism and new therapeutic bioactivities of flavonoids

**DOI:** 10.5599/admet.3404

**Published:** 2026-07-07

**Authors:** Iman Permana Maksum, Tati Herlina, Teruna J. Siahaan, Yaya Rukayadi, Meiske Naomi Mamuaja

**Affiliations:** 1 Department of Chemistry, Universitas Padjadjaran, Bandung, Indonesia; 2 Department of Pharmaceutical Chemistry, The University of Kansas, Kansas, USA; 3 Department of Food Science, Universiti Putra, Selangor, Malaysia; 4 Department Chemistry, Universitas Negeri Manado, Indonesia

**Keywords:** Phenylpropanoid pathway, bioavailability, bioactivity, phytotherapy

## Abstract

**Background and purpose:**

Flavonoids are polyphenolic secondary metabolites synthesized via the phenylpropanoid pathway, serving as a vital biological "immune system" for plants by providing UV-B protection, antimicrobial defence, and pollinator attraction.

**Experimental approach:**

This review examines how these plant-based survival tools translate into potent therapeutic agents for human health through a literature review using Google Scholar, Scopus and ScienceDirect, with keywords alone or in combination with other terms.

**Key results:**

Current research demonstrates that flavonoids possess diverse bioactivities, including antioxidant, anti-inflammatory neuroprotective properties, with significant efficacy in mitigating cardiovascular disease, diabetes and cancer. Despite these benefits, the clinical application of flavonoids is currently hindered by limited bioavailability, which prevents consistent therapeutic concentrations in the human body, and a lack of standardized regulatory dosing.

**Conclusion:**

This paper identifies three critical pillars for future innovation in the development of structural analogs to (a) improve intestinal absorption, (b) explore drug synergy for enhancing conventional medical treatments and (c) evaluate the microbiome interactions to facilitate personalized nutrition. By integrating plant biochemistry with clinical pharmacology, flavonoids can be transitioned from general dietary components into precise, evidence-based therapies.

## Introduction

A large group of phenolic or polyphenol compounds can be defined as flavonoids with one or more aromatic rings along with at least one hydroxyl group, as well as specific additional functional groups [1]. Flavonoids have been recognized in plants for a long time and their function as plant pigments, particularly the anthocyanins, has been the driving force for their early studies [2]. Flavonoids were first isolated in the 1930s from citrus and were initially misclassified as vitamins [3]. Up until the early 1960s, researchers largely considered them mere by-products of plant cell metabolism. However, the application of advanced spectroscopy and analytical techniques, such as nuclear magnetic resonance (NMR) and gas-liquid chromatography (GLC), enabled accurate structural determination, leading to a new appreciation for flavonoids as vital metabolites with significant human health benefits [4]. Since then, an estimated 10,000 or more flavonoid derivatives have been reported. This vast structural diversity is generated by various modification reactions, such as O-methylation catalysed by O-methyltransferases, critically affecting the compound's solubility and bioavailability, factors highly relevant to ADMET studies [5].

Flavonoids perform essential functions in plants, contributing to growth, structure and environmental stress response. Beyond protecting against UV radiation and soil metal toxicity, they are key mediators in the plant's interaction with its ecosystem [6,7]. For instance, their vibrant colours attract pollinators (insects and birds), aiding seed dispersal. The characteristic colours of flavonoids also make it possible for their widespread use in food as a natural colorant, as well as in cosmetics and skincare products [8,9]. The *in vivo* biological activity of flavonoids is influenced by their chemical structure, specifically their degree of hydroxylation, substitution patterns, conjugation and polymerization [10]. Extensive *in vivo* and *in vitro* studies of flavonoids indicated that they have various bioactivities, including antioxidant, cytotoxic, anticancer, anti-inflammatory, antiviral, antibacterial, anti-allergic, antiplatelet, cardioprotective, hepatoprotective, neuroprotective, antimalarial and antiparasitic effects [11-13]. While previous reviews have catalogued the general classes of flavonoids and their bioactivities, this review also examines how these plant-based survival tools translate into potent therapeutic agents for human health and the breakthrough in the *de novo* production.

## Biosynthesis and classification

Flavonoid biosynthesis occurs through the phenylpropanoid pathway; this is a major metabolic route to produce secondary metabolites that support plant growth, structural integrity and responses to environmental stress [14]. This pathway occurs mainly in the cytosol and involves the coordinated action of multi-enzyme complexes; this tight regulation prevents the accumulation of reactive and potentially toxic intermediate metabolites in the cytoplasm [15].

**Figure 1.**
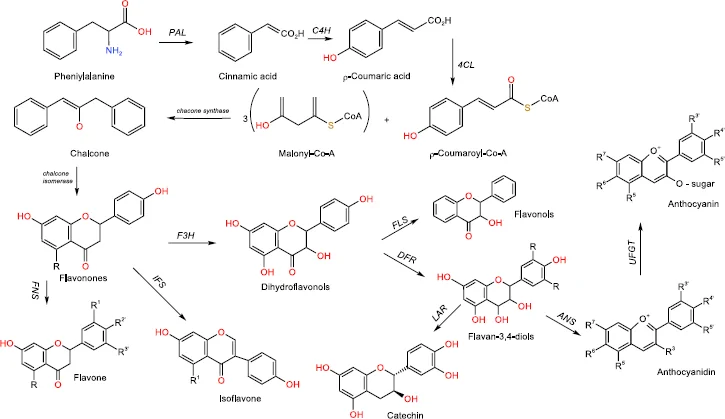
Flavonoid biosynthesis pathway

The biosynthetic pathway (Figure 1) begins with the conversion of phenylalanine to *p*-coumaroyl-CoA through the sequential action of three key enzymes, such as phenylalanine ammonia-lyase (PAL), cinnamate 4-hydroxylase (C4H) and 4-coumarate-CoA ligase (4CL) [16].

This initial step removes the amino group from phenylalanine to produce the phenylpyruvate intermediate. The first enzyme activated in flavonoid biosynthesis is chalcone synthase (CHS). It catalyses the formation of chalcone by condensing one molecule of *p*-coumaroyl-CoA with three molecules of malonyl-CoA [17,18]. As the key precursor, chalcone is then converted to flavanone by chalcone isomerase (CHI) *via* an intramolecular cyclization reaction [19]. CHI enzymes are classified into four types. Type I CHI is present in most land plants and catalyses the conversion of naringenin chalcone into (2S)-naringenin. Type II CHI is specific to leguminous plants and catalyses the formation of 5-deoxyflavanones such as liquiritigenin [20]. Type III proteins resemble CHI structurally but function as fatty acid-binding proteins, while type IV CHI-like proteins (CHIL) assist CHS in chalcone formation [21,22].

Following flavanone formation, flavanone 3-hydroxylase (F3H) converts flavanones to dihydroflavonols as key intermediates for the biosynthesis of flavonols, anthocyanins and proanthocyanidins [23]. Additional hydroxylation reactions on the B-ring are catalysed by cytochrome P450 enzymes such as flavonoid 3′-hydroxylase (F3′H) and flavonoid 3′,5′-hydroxylase (F3′5′H), leading to structural diversity among flavonoids [24]. Different branches of the pathway lead to distinct flavonoid subclasses. Flavone synthase (FNS) transforms flavanones to flavones, while isoflavone synthase (IFS) redirects flavanones into the isoflavone pathway, which is particularly important in leguminous plants [25]. Isoflavone biosynthesis involves several additional enzymes, resulting in a wide range of isoflavone derivatives with diverse biological functions. In some plants, alternative cyclization reactions catalysed by stilbene synthase (STS) produce stilbenes instead of flavonoids. This branch pathway occurs only in specific species such as grapevine, pine, sorghum and legumes. Other specialized enzymes, such as aurone synthase (AURS), generate aurones, yellow pigments commonly found in flowers and involved in pollination [26,27].

After synthesis in the cytosol, flavonoids are transported into the vacuole, where they are stored and stabilized. This transport process requires membrane permeability and tight regulation to ensure vacuolar sequestration and metabolite stability [28]. There are three distinct flavonoid transport mechanisms: vesicle trafficking, membrane transporters and glutathione S-transferase (GST)-mediated transport [29]. In brief, flavonoid transport pathways can be classified as follows [30-32];

1. *Proton-dependent transporters*, in this mechanism, a proton gradient between the cytosol and the vacuole (or cell wall), generated by H^+^-ATPases and H^+^-PPases, is thought to drive flavonoid transport. Once inside the vacuole, the acidic pH and flavonoid acylation promote conformational modification, allowing the formation of stable and functional metabolites.
2. *ATP-binding cassette (ABC) transporters and multidrug and toxic compound extrusion (MATE) transporters:* ABC transporters mediate direct, energy-dependent transport of flavonoids into the vacuole; thus, they prevent the accumulation of potentially toxic intermediate metabolites [26]. These proteins couple ATP hydrolysis with substrate translocation across membranes, often via conjugation with glutathione (GSH) catalysed by glutathione S-transferase (GST). MATE transporters exhibit substrate specificity due to structural mutations. Although their exact mechanism is not fully understood, MATE transporters are believed to play roles in detoxification and disease resistance signalling.
3. *Glutathione S-transferase (GST)-mediated transport:* **I**n this pathway, flavonoids bind to GST as a carrier protein to facilitate their transport to the vacuole. GSTs are also associated with cellular membranes of the endoplasmic reticulum (ER) and vacuole, particularly in plant cells that produce high levels of anthocyanins.

Flavonoids are characterized by a basic 15-carbon flavone backbone (C6-C3-C6) with two benzene rings (A and B) connected by a three-carbon pyran ring. Based on the variations in the C3 unit, flavonoids are further divided into subclasses, including flavanones, isoflavones, anthocyanins, chalcones, dihydrochalcones, flavanols and proanthocyanidins [33]. Furthermore, according to structural modifications of the core skeleton, flavonoids are commonly classified into seven major subclasses (Table 1) [34,35]. In nature, flavonoids occur in two main forms: glycosides and aglycones [36]. Glycosylation in natural products involves the attachment of sugar moieties through carbon-carbon or carbon-oxygen bonds, resulting in more stable metabolites through the interaction between secondary metabolite acceptors and sugar groups [37].

**Table 1.** Classification of flavonoids

Subclass	Key structural feature	Ring B attachment	C2-C3 bond	Common aglycones
Flavones	C4 ketone group; lack of C3 hydroxyl	C2	Double	Apigenin, luteolin
Flavonols	3-hydroxyflavones; hydroxyl group at C3	C2	Double	Quercetin, kaempferol
Flavanones	Dihydroflavones; lack of C2-C3 double bond	C2	Single	Naringenin, hesperetin
Isoflavonoids	B-ring migration from C2 to C3	C3	Double	Genistein, daidzein
Flavanols	Flavan-3-ols; C3 hydroxyl, no C4 ketone	C2	Single	Catechin, epicatechin
Anthocyanins	Flavylium ion (chromenylium) skeleton	C2	Double	Cyanidin, pelargonidin
Chalcones	Open-chain; α, β unsaturated carbonyl	N/A	N/A	Isoliquiritigenin, phloretin

Among secondary metabolites, flavonoid production in plants is highly limited and varies by plant species, plant parts, age and environmental conditions. The very low levels of plant flavonoid production pose a challenge for research on these secondary metabolites. Studies have shown that flavonoid production can be enhanced through several approaches, including triggering production-inducing factors, regulating enzymes involved in flavonoid biosynthesis and utilizing microorganisms[38-40]. Plant metabolic engineering is carried out through the overexpression of key enzymes that actively participate in flavonoid biosynthetic pathways [41]. In addition, regulation of transcriptional and translational mechanisms using transcription factors, target gene expression [42] and prevention of end-product toxicity in plants [43] can also be implemented. Furthermore, the use of microorganisms for flavonoid production has gained considerable attention, particularly because of its low energy requirements, high product purity and low emissions of waste such as sulfates, nitrates, or nitrites [44]. For example, microorganisms such as Escherichia coli and Saccharomyces cerevisiae can produce naringenin and apigenin [45-47]. The *de novo* production of flavonoids by microorganisms can yield certain flavonoids at gram-per-liter fermentation levels, which are significantly higher than those in natural plant production. For comparison, the naringenin content in grapefruit (*Citrus paradisi*) was 16.90 mg *per* 100 mg [48], whereas the use of *Corynebacterium glutamicum* was able to produce 35 mg L^-1^ naringenin and 37 mg L^-1^ eriodictyol [49] and *Escherichia coli* produced 155 mg L^-1^ naringenin [50].

## Biological functions of flavonoids in plants

### Protection against abiotic stress

Flavonoids are key secondary metabolites that enable plants to overcome abiotic stress through their structural diversity and antioxidant properties. Abiotic stresses activate cellular signaling pathways that influence plant reactions to ultraviolet (UV) radiation, drought, salinity, extreme temperatures, heavy metal toxicity and nutrient deficiency [51-55]. Under high salinity and drought conditions, plants enhance flavonoid accumulation to mitigate oxidative damage. For example, NaCl treatment increases flavonoid and flavonoid glycoside levels in *Ginkgo biloba* seeds and activates flavonoid-based antioxidant systems in *Casuarina glauca*, improving salt tolerance [56,57]. Cold stress also induces flavonoid production; increased phenolics and flavonoids have been reported in tomato, sorghum, *Haberlea rhodopensis* and *Arabidopsis thaliana*, where flavonols and anthocyanins protect membranes and proteins from freezing damage [58-60].

Excessive UV radiation produces oxidative stress and DNA damage in plants. As a defence, plants accumulate UV-absorbing flavonoids such as quercetin and luteolin glycosides, which act as effective antioxidants and UV screens [61,62]. UV-B exposure strongly induces flavonoid biosynthesis and upregulates key genes such as chalcone synthase (CHS), as demonstrated in apple, tomato, *Ligustrum vulgare*, *Isatis tinctoria* and soybean [63-67]. Heavy metal stress stimulates flavonoid production, which contributes to metal chelation and reduces oxidative damage. Increased flavonoid accumulation and enhanced expression of phenylpropanoid-related enzymes have been observed in plants exposed to Cu, Cd, Cr, Pb and Se [68,69].

Nutrient imbalance is another major abiotic stress. Under nutrient-deficient conditions, plants release flavonoids into the rhizosphere via ABC transporters to improve nutrient availability [70]. Isoflavones and flavonols facilitate iron and phosphate uptake by reducing Fe^3+^ to Fe^2+^ and chelating metal ions [71,72]. Deficiencies in nitrogen and phosphorus also induce the expression of flavonoid and anthocyanin biosynthetic genes, leading to increased antioxidant capacity in crops such as red and green cabbage [73,74].

### Pigmentation and pollinator attraction

Insect pollination (entomophily) is the primary reproductive strategy of flowering plants, in which pollinators identify flowers mainly through visual cues such as colour and, to a lesser extent, the floral scent [75]. Flower colour signals the reproductive status of plants and provides information on nectar and pollen availability, thereby acting as a key visual attractant for pollinators [76]. The structural diversity of flavonoids enables the production of a wide range of flower colours that match pollinator visual perception [77]. Pollinators exhibit species-specific preferences influenced by colour, scent and reward availability, with some insects showing strong attraction to yellow hues due to their dichromatic visual systems [78,79]. Anthocyanins are the dominant flavonoid pigments involved in pollinator attraction and accumulate in flowers and fruits. Six anthocyanidins, delphinidin, pelargonidin, cyanidin, petunidin, peonidin and malvidin, are widely distributed in edible plants [80]. Differences in hydroxylation patterns and pH conditions generate colours ranging from red to blue, with increased hydroxylation and higher pH favouring bluish hues [81]. Floral colour evolution is closely linked to pollinator perception of specific pigment combinations. Anthocyanins create distinct colour loci that are consistently perceived across diverse pollinators, including bees, butterflies, mosquitoes and birds [82].

### Regulation of plant growth and development

In sexual reproduction of flowering plants, male gametes contained in pollen grains are delivered to the female gametophyte through pollen tube formation, which grows through the pistil to fertilize the ovule [83]. Studies in *Petunia hybrida* showed that transgenic plants with blocked flavonol biosynthesis (via CHS inhibition) lacked flavonols in the stigma, ovules, pollen and pollen tubes, resulting in male sterility [84]. Although pollen initially germinated normally, *in vitro* assays revealed impaired pollen tube growth, protoplasmic damage, and eventual pollen death. Similar results were reported in maize and petunia mutants deficient in CHS, where pleiotropic effects disrupted pollen fertility and flavonoid biosynthesis [85]. Supplementation with kaempferol restored pollen germination and pollen tube growth *in vitro* and increased seed set *in vivo*. Likewise, anthocyanin-deficient tomato mutants exhibited reduced seed production due to decreased pollen quantity, viability, germination and pollen tube elongation [86]. This phenotype was associated with elevated ROS and H_2_O_2_ accumulation in pollen and pollen tubes, reflecting the loss of flavonols as ROS scavengers in reproductive tissues. In rice, mutant studies demonstrated that multiple flavonoid classes, including flavanones, flavonols, flavones, and their glycosides, are required for anther fertility and male reproduction, in contrast to other plant species that rely predominantly on flavonols alone [87].

Flavonoids also play a role in the regulation of the plant hormone auxin, where auxin and cytokinin act synergistically or antagonistically to control development and modify growth in response to environmental cues [88]. The activity of P-glycoproteins that transport auxin is modulated by flavonoids, and they influence phosphatases and kinases as regulatory proteins [89]. Auxin activity increases in response to reduced water availability and suboptimal soil mineral nutrient levels. The evolution of auxin transport mechanisms is closely associated with the production of flavonoids, which function to counteract reactive oxygen species and provide defence against herbivores and pathogens [90].

### Symbiotic signalling and defence

The rhizosphere serves as a dynamic habitat for soil microorganisms that suppress pathogen invasion and enhance plant nutrient acquisition [91]. Flavonoids play a key role in rhizosphere signalling by stimulating rhizobial chemotaxis, promoting bacterial colonization and inducing nodulation (nod) gene expression [92]. Under unfavourable environmental conditions, plants actively release flavonoids and other metabolites into the rhizosphere through root exudation to initiate beneficial plant-microbe interactions [93]. Once released, flavonoid activity depends on soil properties and structural modifications. Flavonoids may bind to soil particles and become inactive, while glycosylated forms are rapidly deglycosylated by microbes to yield more hydrophobic aglycones [94]. In addition, several flavonoids act as quorum-sensing inhibitors (QSI), influencing bacterial biofilm formation, nitrogen fixation and motility; examples include catechin and naringenin, which also function as nod gene inducers in legumes [95,96].

Allelopathy refers to a form of plant interference in which defence metabolites (allelochemicals) are produced and released to negatively affect neighbouring plants [97]. Flavonoids function as allelochemicals exuded by roots, inhibiting seed germination by inducing reactive oxygen species (ROS). For example, isoschaftoside, a C-glycosyl flavone isolated from *Desmodium uncinatum*, suppresses the growth of the hemiparasitic weed *Striga hermonthica* that damages maize [98]. Similarly, catechin released from the roots of *Centaurea maculosa* inhibits germination and growth of native Montana plant species, where older plants may be more resistant [99].

## Flavonoid absorption and metabolism

In general, flavonoids are bound in plant cells through hydroxyl (OH) groups as O-glycosides *via* O-glycosidic bonds or as C-glycosides through carbon-carbon (C-glycosidic) bonds [100,101]. In C-glycosides, the glycosidic bond is directly attached to the flavonoid backbone, typically at the C-6 or C-8 positions of the A-ring, resulting in compounds that are more resistant to hydrolysis and consequently exhibit altered biological activity. Hydrophilic glycoside forms can be absorbed in the small intestine mainly through passive diffusion after conversion to aglycones [102].

The physicochemical properties of flavonoids can influence the oral absorption of dietary flavonoids; these properties include molecular size and structure, solubility, lipophilicity and acidity [103]. Therefore, the flavonoid content in the food is less important than the fraction of the compound that is orally bioavailable [104]. Before absorption, flavonoids must be released from the food matrix through mastication, enzymatic digestion in the gastrointestinal tract and microbial metabolism in the intestine [105]. Flavonoid glycosides that are not absorbed in the small intestine are converted into aglycones in the cecum and colon by intestinal microbiota via methylation, sulfation and glucuronidation [106]. Lactase-phlorizin hydrolase (LPH) hydrolyses flavonoid glycosides into aglycones, which are subsequently absorbed by passive diffusion. LPH is a mammalian β-glycosidase located on the brush-border membrane of the intestine and is specific for flavonoid O-β-D-glucosides [107]. *In vitro* studies using LPH purified from sheep small intestine demonstrated that flavonoid glycosides serve as substrates for this enzyme [108]. The specificity of LPH substrates varies depending on the glycoside type (glucoside, galactoside, arabinoside, xyloside, rhamnoside, or rutinoside) and the flavonoid subclass, including flavones, isoflavones, flavonols, flavanones and anthocyanins [109]. In individuals with lactase deficiency, for example, reaches 87 % in three major ethnic groups in Malaysia, plasma isoflavone levels are initially low during early absorption but later become comparable to those of lactase-sufficient individuals, likely due to compensatory microbial hydrolysis in the gut [110,111]. Deglycosylation of flavonols is highly efficient, as glycoside forms are undetectable in plasma after administration of quercetin glycosides [112]. In contrast, anthocyanins can resist deglycosylation, as evidenced by their presence in urine [113].

Another enzyme involved in glycoside hydrolysis is cytosolic β-glucosidase (CBG), which is specific for 7-O-glucosides [114]. *In vitro* studies using human liver and small intestine tissues demonstrated that CBG acts on isoflavone, flavone and flavanone-7-glucosides but not on naringin, which contains a 7-rhamnoglucoside moiety [115]. Hydrolysis products are subsequently transported by sodium-glucose transporter 1 (SGLT1) and distributed via passive transport into the portal vein or metabolized through phase I and II reactions [116]. Together with GLUT2, SGLT1, widely expressed in intestinal epithelium, is involved in the detection and absorption of flavonoid glycosides from the food matrix [117].

Glycosides that are not substrates for LPH or CBG reach the colon, where they are hydrolysed and degraded into aglycones [118]. For example, the bioavailability of quercetin-3-β-rutinoside in human volunteers was only 20% compared to quercetin-4′-β-glucoside, indicating that sugar moieties strongly influence absorption and bioavailability. Food processing methods, such as fermentation and autolysis, can cleave flavonoid glycosidic bonds [119]. Fermented soybean products containing flavonoid aglycones are more readily absorbed than non-fermented soybeans, due to the transformation of flavonoids into glucosides, sulfonyl conjugates and glucuronides by the microbes [120].

Flavonoids in aglycone form are directly absorbed in the small intestine via passive diffusion due to increased lipophilicity and are transported to the hepatic vein, influenced by lipid solubility and molecular interactions; thus, dietary fat content affects flavonoid bioavailability [116]. In rat studies using lymph duct cannulation, administration of quercetin with long-chain fatty acids significantly increased hepatic quercetin levels compared to combinations with glucose or medium-chain fatty acids [121]. These findings suggest that long-chain fatty acids enhance quercetin bioavailability by promoting lymphatic transport and bypassing hepatic phase I metabolism. The addition of milk to black tea does not alter the absorption curves of flavonols and catechins, indicating that proteins do not affect flavonoid absorption [102]. In contrast, alcohol significantly enhances flavonoid absorption. Studies using rat intestinal sac models showed a threefold increase in quercetin absorption and a 1.5-fold increase in quercetin-3-O-glucoside absorption when combined with alcohol [122]. In human volunteers consuming alcohol with catechins, plasma catechin levels increased threefold within one hour and were present as distinct metabolites [123].

After intestinal absorption, flavonoids undergo extensive first-pass metabolism mainly in the liver and colon. In the liver, Phase I reactions, primarily oxidation or O-demethylation mediated by cytochrome P450 enzymes, are followed by Phase II conjugation catalysed by UGTs, SULTs and COMT, yielding glucuronidated, sulphated, or methylated metabolites [124,125]. Unabsorbed flavonoids and conjugated metabolites excreted via bile enter the colon, where gut microbial enzymes deconjugate them into aglycones that may be reabsorbed through enterohepatic circulation or further degraded [126]. The metabolic fate of flavonoids is strongly influenced by structural features, as highly hydroxylated compounds are more susceptible to microbial degradation, whereas O-methylation enhances metabolic stability [127]. In systemic circulation, flavonoids predominantly bind to serum albumin, with binding affinity governed by hydroxylation patterns and glycosylation [128]. Transport into tissues occurs through regulated membrane transporters and passive diffusion, depending on molecular size and hydrophobicity, while highly hydrophobic aglycones preferentially enter the intestinal lymphatic system, facilitating their distribution to peripheral tissues [129-131].

## Therapeutic bioactivities of flavonoids

### Antioxidant activity

Antioxidant activity is the biological property most commonly associated with flavonoids. As antioxidants, flavonoids act as free radical scavengers that neutralize reactive oxygen species (ROS), which cause cellular damage and contribute to various diseases, such as cardiovascular disorders, neurodegenerative disorders and cancer [132]. Flavonoids exert their antioxidant effects through multiple mechanisms, including direct scavenging of ROS [133], activation of endogenous antioxidant enzymes [29], metal ion chelation [134], inhibition of xanthine oxidase and NADPH oxidase [135], suppression of nitric oxide-mediated oxidative stress [136] and enhancement of low-molecular-weight antioxidants [137]. The hydroxyl groups of flavonoids, particularly ortho-dihydroxyl groups on the B-ring, play a crucial role by donating hydrogen atoms and electrons to stabilize free radicals [138]. Structural features such as the number and position of hydroxyl groups, conjugation and planarity strongly influence antioxidant potency [139].

Several flavonoid activities result from a synergistic combination of radical scavenging and enzymatic modulation [140]. Flavonoids can activate key defence proteins, including NAD(P)H: quinone oxidoreductase, glutathione S-transferase and UDP-glucuronosyltransferase, which serve as the primary defence line against electrophilic toxins and oxidative stress [141]. For instance, onion extract and quercetin can increase intracellular glutathione (GSH) concentrations by approximately 50 % [142]. This occurs through the up-regulation of γ-glutamylcysteine synthetase (GCS), the rate-limiting enzyme in GSH synthesis. GSH acts as a major cellular antioxidant, maintaining redox equilibrium, while GSH peroxidase facilitates the clearance of various ROS and reactive nitrogen species (RNS) [143].

Flavonoids can chelate or bind metal ions to prevent metal-induced oxidation. The ability of flavonoids to form stable complexes with metal ions is dictated by specific structural features within the molecule. Primarily, there are three potential binding sites for metal ions: the 3',4'-dihydroxy (catechol) group on the B-ring and the 3-hydroxy or 5-hydroxy groups in conjunction with the 4-carbonyl group on the C-ring [144]. Through these hydroxyl and carbonyl groups, a wide array of plant-derived flavonoids can sequester transition metals to prevent metal-induced oxidation. For example, epigallocatechin-3-gallate (EGCG), the primary constituent of green tea, representing over 50 % of its catechins, can effectively inactivate Cu(II) by chelating Cu(I), subsequently forming peroxymonosulfate species with higher oxidative activity than Fe(II) [145]. However, the outcome of these interactions is highly dependent on the local environment and concentration. Research indicates that the shift between antioxidant and pro-oxidant behaviour is governed by the concentration of both the flavonoid and the metal ions involved [146]. In practical applications such as canned foods, quercetin has been shown to form a quercetin-Tin(II) complex that simultaneously reduces the antioxidant capacity of the flavonoid and the concentration of tin [147]. The studies have shown that while Fe and Zn possess high pro-oxidant effects, inducing 37 and 33 % haemolysis, respectively, they can be successfully chelated by quercetin, rutin and catechin. Notably, this protective chelation effect appears markedly less significant against Zn ions compared to Fe [146].

Flavonoids function as potent regulators of oxidative stress through the targeted inhibition of key oxidase enzymes, most notably xanthine oxidase (XO) and NADPH oxidase (NOX) [148]. Their inhibitory efficacy is strictly governed by specific structure-activity relationships (SAR), where molecular planarity and the strategic positioning of hydroxyl groups—particularly at the C-5 and C-7 positions of the flavonoid skeleton—are essential for high-affinity binding to enzyme active sites. By blocking these enzymatic pathways, flavonoids prevent the catalytic production of harmful reactive species, such as hydrogen peroxide and superoxide anions. Beyond direct competitive inhibition, flavonoids like quercetin also offer a secondary layer of protection by inducing cytoprotective enzymes such as heme oxygenase-1 (HO-1), thereby suppressing NOX-mediated oxidative damage [149]. This dual action, acting as both direct inhibitors of pro-oxidant enzymes and indirect stimulators of cellular defence systems, positions flavonoids as critical therapeutic candidates for mitigating the oxidative pathologies associated with chronic conditions like diabetes and inflammatory disorders [150].

### Antidiabetic activity

Flavonoids exhibit potent anti-diabetic properties by modulating glucose homeostasis, enhancing insulin sensitivity and protecting pancreatic function through multiple molecular pathways (Table 2). Each class of flavonoids exhibits diverse antidiabetic properties, including enhancing insulin secretion and pancreatic β-cell viability under high-glucose or pro-inflammatory conditions. Therefore, they improve insulin-stimulated glucose uptake in target cells, protecting muscle cells from fatty acid-induced insulin resistance and reducing hyperglycemia while improving glucose tolerance in animal models of obesity and type 2 diabetes mellitus [151]. Table 2 summarizes studies on the antidiabetic effects of several flavonoids.

**Table 2.** Antidiabetic effects of several flavonoids.

Mechanism of Action	Flavonoid
Inhibits α-glucosidase and α-amylase	Luteolin [152], chrysin, salvigenin [153], nepetin [154], epigallocatechin gallate [155], catechin [156], anthocyanin [157]
Increases insulin secretion	Luteolin [158], salvigenin [159], rutin [160], epicatechin [161], genistein [162]
Reduces fasting blood glucose and HbA1c	Luteolin [163], quercetin [164], rutin [165,166], fisetin [167], catechin [168], daidzin and glycitin [169], daidzein [170], biochanin [171], formononetin [172]
Strengthens glucose-stimulated insulin secretion (GSIS)	Apigenin [173], epicatechin [174]
Protects beta-cells	Apigenin [175], kaempferol [176], quercetin [177], isorhamnetin [178], morin [179], epigallocatechin gallate [180], anthocyanin [181], genistein [182], equol [183], formononetin [184]
Enhances GLUT4 translocation	Acacetin [185], isorhamnetin [186], equol [187], formononetin [188], naringenin [189]
AMPK activation	Nepetin [190], quercetin [191], anthocyanin [192]. daidzein [193], naringenin [194]
Improves insulin resistance	Isorhamnetin [195,196], epigallocatechin gallate [197], epicatechin [198], anthocyanin [199], biochanin a [200], hesperetin and hesperidin [201]

### Anticancer activity

The anticancer activity of flavonoids is linked to the ability of this group of compounds to interfere with key processes in cancer development (Table 3) [202]. The mechanisms of action can be divided to [203-207]:

- Interference with signalling pathways, by disrupting signal cascades such as MAPK, PI3K/Akt that regulate cancer cell growth and proliferation.
- Suppression of inflammatory mediators, for example, by inhibiting NFκB, thereby reducing chronic inflammation that often promotes tumour progression.
- Structural inhibition, flavonoids specifically target processes that enable tumours to form new blood vessels (angiogenesis) and spread to distant organs (metastasis).
- Induction of programmed cell death, flavonoids can trigger apoptosis, a natural cellular self-destruction mechanism that is frequently dysregulated in cancer cells.

**Table 3.** Anticancer activity of several flavonoids

Mechanism of action	Flavonoid
Inhibits cell proliferation	Apigenin [208-210]
Reducing chemoresistance and drugs toxicity	Apigenin [211], scutellarin [212], salvigenin [213]
Induces apoptosis	Apigenin [214], acacetin [215], pectolinarigenin [216,217], luteolin [218], luteolin [219,220], nepetin [221], scutellarin [222], hispidulin [223], hesperetin cells [224]
Reduces cell proliferation by modulating the MAPK	Apigenin [225,226]
Regulates the production of pro-inflammatory cytokines (TNF-α, IL-6, IL-8, *etc*.)	Apigenin [227], hispidulin [228]
Suppresses cytoplasmic transcription factor (STAT3)	Apigenin [229], pectolinarigenin [230], luteolin [231], salvigenin [232]
Downregulates PI3K/AKT/mTOR pathway	Apigenin [233], pectolinarigenin [234], nepetin [235], scutellarin [236], hispidulin [237,238]
Inhibits cell proliferation, migration and invasion	Luteolin [239], nepetin [240], salvigenin [241], hesperetin [242,243]
Inhibitors of the transforming growth factor-β (TGF-β)	Hesperetin [244]

### Anti-inflammatory

Flavonoids have been extensively researched as anti-inflammatory agents, which is linked to their planar structural conformation featuring an unsaturated bond at C2-C3, as well as the critical position of the hydroxyl groups for this property [245]. The flat shape of the molecule allows it to fit more effectively into the binding pockets of inflammatory enzymes like COX-2 or iNOS [246]. Research indicates that flavonoids downregulate pro-inflammatory cytokine expressions, including TNF-α, IL-1β, IL-6, IL-8 and MCP-1 in RAW macrophage cells, peripheral blood mononuclear cells and Jurkat T cells [247]. Flavonoids are also effective in decreasing pro-inflammatory enzymes such as iNOS, COX-2, glucuronidase and lysozyme, while enhancing antioxidant enzymes like GST, heme-oxygenase-1, SOD and CAT, which play vital roles in the progression of inflammatory diseases [248]. The presence of hydroxyl groups at the 3' and 4' positions of the B ring is responsible for the anti-inflammatory activity of the flavonoid group [249,250].

Apigenin, a flavone, has been reported to lower TNF-α-induced steady-state mRNA levels, thereby reducing the expression of ICAM-1, E-selectin and VCAM-1 in endothelial cells [251]. Flavonols like quercetin, morin, kaempferol and myricetin act as lipogenesis inhibitors and potentially inhibit arachidonic acid, phospholipase A2, cyclooxygenase and NOS, thus lowering the production of prostaglandins, leukotrienes and NO, which are key inflammatory compounds [252]. Catechin and quercetin were also reported to increase the production of IL-10, an anti-inflammatory compound, while simultaneously inhibiting IL-1β and TNF-α [251]. Quercetin blocks heat shock factor (HSF) activity required to induce the HSP70 protein, thereby reducing heat-induced damage [253]. Additionally, flavonoids can chelate iron and inhibit complement system activation to decrease inflammation [254].

Hispidulin increases dopamine in the prefrontal cortex of phencyclidine-induced rats and reverses "anti-social" behaviour in schizophrenia-1 mutant rats [255]. In epilepsy models, hispidulin reduces seizures with effects comparable to diazepam and alleviates motor disorders in hyper-dopamine-stressed rat models [256,257]. Its anti-seizure effects work by suppressing inflammatory processes and activating MAPK-A [258]. Acacetin has been reported to have anti-neuroinflammatory activity in Parkinson's disease models by protecting dopaminergic cells and inhibiting inflammatory factors like NO, prostaglandin E2 and TNF-α [259]. In dental inflammation, acacetin suppresses inflammation by regulating autophagy and GSK-3β signaling in human periodontal ligament cells [260]. Luteolin inhibits neuroinflammation and reduces endoplasmic reticulum stress markers in brain tissue [261]. In subarachnoid haemorrhage rat models, it improves oxidative damage by increasing Nrf expression and downregulating NLRP3 activation [262]. For intracerebral haemorrhage, it protects against microglia activation and infiltration and inhibits the TLR4/TRAF6/NF-κB signalling pathway [263].

Scutellarin protects against vascular inflammation induced by hyperglycaemia and inhibits testicular apoptosis and morphological disorders in diabetic rats [264]. Scutellarin protects against intervertebral disc degeneration (IVDD) by reducing ROS and mitochondrial damage [265]. In SARS-CoV-2 simulations, it interacts with the ACE2 receptor with a binding energy of -62.3415 kJ/mol at Glu495, Unk957 and Arg482 residues [266]. In stroke therapy, scutellarin aids in brain ischemia by activating JAK2/STAT3 signalling [267], and reduces brain tissue infarction in MCAO rat models [268]. Hesperidin and hesperetin prevent viral binding to ACE2, inhibit replication, and neutralize excessive pro-inflammatory reactions in SARS-CoV-2 infections [269]. Molecular docking shows hesperidin binding affinities for SARS-CoV-2 Mpro, SARS-CoV-2 PLpro, and spike glycoprotein are -24.27, -41.84 and -33.89 kJ mol^-1^, respectively [270]. It also has a high affinity for targets, preventing viral RNA synthesis (3CLpro and Helicase) and binds the spike-ACE2 interface via hydrogen bonding with the Tyr440 residue [271].

### Cardiovascular protection

Cardiovascular disease (CVD) conditions can be categorized into atherosclerosis, hypertension, and cardiomyopathy [272]. In these cases, the cardiovascular protective effects of flavonoids have been reported across all three conditions [273]. The protective effect of flavonoids against cardiovascular disorders occurs through several specific mechanisms, primarily targeting hypertension, atherosclerosis and cardiomyopathy.

Hypertension is categorized into primary hypertension, which results from hyperlipidemia, insulin resistance and obesity, and secondary hypertension, which refers to high blood pressure caused by kidney failure, vascular disease (narrowing of blood vessels) and endocrine disorders [274]. Flavonoids lower the occurrence of hypertension through several key mechanisms.

First, flavonoids can act as vasorelaxants by directly modulating vascular smooth muscle and endothelial signalling pathways. Several flavones (acacetin, apigenin, luteolin, chrysin) and flavanones (hesperetin, pinocembrin) induce endothelium-dependent and -independent vasorelaxation, primarily via activation of Ca^2+^-activated potassium channels and enhancement of nitric oxide (NO) bioavailability [275-277]. Quercetin improves vascular function by restoring endothelial NO production and nitric oxide synthase (NOS) activity under hypertensive conditions [278]. Similarly, naringenin promotes vasorelaxation by activating K-Ca^2+^ channels in vascular myocytes and reduces blood pressure and cardiac hypertrophy through regulation of the AMPK/NOX2/MAPK signalling pathway [279]. In addition, hesperetin-7-O-β-glucuronide enhances NOS activity and attenuates oxidative stress in the aorta, further contributing to its vasorelaxant effects [280].

Second, flavonoids can suppress oxidative stress that induces endothelial dysfunction in the early event of atherosclerosis [281] and flavonoids improve endothelial function by directly enhancing NO-mediated vasoprotection and suppressing vascular inflammation [282]. They mitigate this process by preventing the reaction between nitric oxide (NO) and reactive oxygen species (ROS), thereby reducing the formation of the vasoconstrictive and cytotoxic species peroxynitrite (ONOO^-^) [283]. Compounds such as hesperidin and baicalin enhance endogenous antioxidant defences by increasing superoxide dismutase (SOD) and glutathione (GSH) activity while lowering malondialdehyde (MDA) levels [284,285]. In addition, luteolin attenuates vascular hypertension by inhibiting angiotensin II-induced vascular smooth muscle cell proliferation and migration through suppression of MAPK signalling and ROS generation [286]. Luteolin rapidly activates nitric oxide synthase (NOS), increasing NO production and inducing vasorelaxation in rat aortic rings and primary human aortic endothelial cells [287]. In parallel, quercetin metabolites inhibit endothelial adhesion molecule expression (VCAM-1 and ICAM-1), thereby reducing monocyte adhesion and preserving vascular homeostasis [288]. Flavonoids have been found to lower blood pressure by modulating calcium signalling in vascular smooth muscle. Epigallocatechin-3-gallate and hesperetin inhibit voltage-operated Ca^2+^ channels, reducing intracellular Ca^2+^ levels and ROS production [289], while genistein suppresses Ca^2+^-dependent proline-rich tyrosine kinase 2 signalling [290].

Third, they can modulate the overactivation of the renin-angiotensin-aldosterone system (RAAS), which is a major cause of hypertension. The inhibition of the RAAS system *via* suppression of angiotensin-converting enzyme (ACE) reduces the formation of the vasoconstrictor angiotensin II [291-293]. Flavonoids such as quercetin inhibit ACE, likely by chelating the active-site zinc ion, and significantly reduce ACE activity *in vivo* [294-296].

Fourth, the anti-inflammatory effects of flavonoids suppress persistent inflammation that leads to stroke and heart failure [297]. Flavonoids modulate the NF-κB and MAPK pathways, lowering the expression of TNF-α, IL-1β and COX-2 [298]. The gut microbiota ferment flavonoid glycosides into short-chain fatty acids (SCFAs) and phenolic metabolites that enter the circulation and modulate inflammation and vascular function [299,300]. For example, quercetin, metabolized by gut microbes to form 3-(3-hydroxyphenyl) propionic acid, inhibits monocyte adhesion induced by TNF-α [301].

The anti-atherosclerotic mechanisms of flavonoids related to vascular health include inhibition of low-density lipoprotein (LDL) oxidation, anti-platelet activity, reduction of atherosclerotic lesions, lowering of blood pressure and improvement of both endothelial and vascular smooth muscle function [302,303]. Flavonols and flavan-3-ols are the most extensively researched flavonoid groups regarding atherosclerotic effects because they are abundant in food and share structural similarities, specifically the presence of a hydroxyl group at C3, with differences occurring in the carbonyl group and the double bond in flavonols [304]. For example, quercetin and theaflavin significantly reduce atherosclerotic lesion size in ApoE-deficient mouse models [305], while glabridin reduces LDL oxidation [306]. In addition, myricetin protects against endothelial vascular cell damage and inhibits atherosclerotic plaque formation in ApoE-deficient mice [307]. Kaempferol has been shown to reduce the atherosclerotic lesion area, improve endothelium-dependent vasorelaxation, increase maximum relaxation values and decrease pro-inflammatory cytokines in ApoE-deficient mice [308].

Several studies indicate that the protective effects of flavonoids in cardiomyopathy occur through the modulation of autophagy, moving in the opposite direction of the triggers that cause cardiomyopathy [309]. *In vivo* research on hypertrophic cardiomyopathy (HC) rat models (induced by isoproterenol-ISO injection) showed that baicalein significantly attenuates HC conditions and restores cardiac function by increasing the expression of catalase and the mitophagy receptor FUNDC1 [310]. Diosmetin, a flavone, was reported to protect against HC under pressure overload via the p62/Keap1/Nrf2 signalling pathway in male C57BL/6 mice [311]. An *in vitro* study using rat cardiomyocyte cultures induced with polysaccharides showed that luteolin can decrease the expression of HC markers, such as atrial natriuretic peptide (ANP) and brain natriuretic peptide (BNP), as well as reduce α-actinin and LC3 expression [312]. Furthermore, *in vitro* tests on H9c2 cells showed that treatment with puerarin, 24 hours before ISO induction, reduced hypertrophic and apoptotic markers, while the *in vivo* results in Sprague-Dawley rats showed improved left ventricular function and a decrease in HC markers and cardiomyocyte apoptosis following the administration of 100 mg *per* kg body weight of puerarin [313].

Isoflavones are a particularly interesting group of flavonoids regarding cardiovascular protection. As phytoestrogens, these soy-derived flavonoids improve cardiovascular risk through several mechanisms. Ecological research found that the high consumption of soy containing isoflavones in Asian countries lowers the risk of cardiovascular disease [314]. A study of 200 early-menopausal Caucasian women (average age 55) who were given 15 g of soy protein containing 66 mg of isoflavones for six months showed improvement in cardiovascular risk markers compared to those given soy protein without isoflavones [315]. Genistein directly affects NOS enzyme activity in vascular endothelial cells, leading to increased NO synthesis [316] and daidzein exhibits vasodilatory properties by stimulating prostaglandin production [317].

### Anti-aging

Skin aging is triggered by intrinsic factors related to genetics and age and extrinsic factors related to environmental exposure; both lead to a decline in structural integrity and a loss of physiological skin function [318]. The mechanism of extrinsic skin aging involves the formation of ROS and oxidative stress caused by environmental factors like UV radiation, cigarette smoke and pollutants [319]. ROS and oxidative stress stimulate the upregulation of MMP (matrix metalloproteinases) and elastase (enzymes responsible for skin elasticity), resulting in the degradation of collagen and elastin in the skin matrix [320]. Tyrosinase is a critical enzyme in melanin biosynthesis across mammals, bacteria, plants and fungi. It is a copper-containing bifunctional enzyme that catalyses the hydroxylation of monophenols into *O*-diphenols and the oxidation of *O*-diphenols into *O*-quinones, which form melanin [321]. Excess melanin production leads to dermatological disorders such as age spots, melasma, freckles, lentigo, brown spots and even skin cancer [322].

Research has reported the ability of flavonoids to inhibit melanin formation reactions through targets such as microphthalmia-associated transcription factor (MITF), tyrosinase, tetratricopeptide repeat (TPR)-1 and TPR-2 [323]. Inhibiting tyrosinase by blocking its active site and preventing its natural substrate, tyrosine, from binding can suppress melanin production, making it a target for hyperpigmentation therapy [324]. Some flavonoids were reported to act as tyrosinase inhibitors. Multiflorin B, a flavone glycoside isolated from *Rosa chinensis*, exhibits tyrosinase inhibitory activity twice as strong as α-arbutin [325]. Tricin, an O-methylated flavonoid, shows higher effects than arbutin, with molecular docking showing hydrogen bonds with Asn80 and Arg267 near the catalytic core [326].

Four flavonoids isolated from *Loranthus acutifolius* show anti-tyrosinase and anti-melanin activities in an *in vitro* study using B16-F10 cells, with the IC50 value lower than 10 μM [327]. Apigenin possesses skin-whitening activity through its 7 and 4’ hydroxyl groups [328]. It forms hydrogen bonds with Met280 and hydrophobic interactions with Val248, Phe264 and Phe292 [329]. In vivo studies show it significantly inhibits hydroquinone-induced vitiligo by acting as an anti-inflammatory and modulating p38 MAPK [330]. Quercetin inhibits tyrosinase monophenolase and diphenolase activities [331]. Its catechol structure (3’,4’-dihydroxy group on the B-ring) chelates copper (Cu) at the tyrosinase active site, blocking the substrate L-DOPA. Quercetin-7-O-α-L-rhamnoside inhibits tyrosinase activity and melanogenesis in B16F10 melanoma cells stimulated by α-MSH plus IBMX. In addition, molecular docking simulations show the formation of hydrogen bonds between this flavonoid and the His85, His244, Thr261 and Gly281 residues of the tyrosinase [332].

Luteolin 5-O-β-D-glucopyranoside isolated from *Cirsium japonicum* var. maackii acts as a potent competitive inhibitor of L-tyrosine [333]. The 4’ hydroxyl group on the aglycone forms a strong hydrogen bond with the peroxide ion between two Cu ions at the active site, while the B-ring hydroxyls bind to Cys83. Luteolin was reported to inhibit cellular melanogenesis to an extent equivalent to arbutin in B16F10 murine melanoma cells that are stimulated by α-melanocyte-stimulating hormone (α-MSH) [334]. Pectolinarigenin has anti-melanogenesis activity by inhibiting the expression of MITF and tyrosinases (*i.e.* TRP-1 and TRP-2) [335]. Hesperetin has skin-protective activity through *in vitro* research using B16-F10 murine melanoma cells, where hesperetin stimulates melanogenesis via MAPK activation, phosphorylation of CREB and GSK-3β [336]. However, other studies indicate that hesperetin also acts as a competitive tyrosinase inhibitor, as demonstrated by inhibitory kinetics and molecular docking analyses predicting binding at Met280, His61, His85 and His259 [337].

## Conclusions

Flavonoids originate from the phenylpropanoid pathway, a complex biosynthetic process through which plants convert simple amino acids into protective secondary metabolites. In plants, flavonoids play essential roles in stress protection, pigmentation, growth regulation and defence, underscoring their ecological and physiological importance. Upon dietary intake, flavonoid bioavailability is strongly influenced by their chemical structure, glycosylation pattern, food matrix and interactions with intestinal enzymes and gut microbiota, which collectively determine absorption, metabolism and systemic distribution. Despite generally low oral bioavailability, extensive biotransformation generates bioactive metabolites that contribute significantly to their biological effects. These processes underpin the wide range of therapeutic bioactivities attributed to flavonoids, including antioxidant, anti-inflammatory, antidiabetic, anti-aging, anticancer and cardioprotective activities, highlighting their relevance as multifunctional bioactive compounds that bridge plant metabolism and human health. By integrating plant biochemistry with clinical pharmacology, flavonoids can be transitioned from general dietary components into precise, evidence-based therapies.

## Challenges and perspective

Future research must focus on seamlessly integrating flavonoid biosynthesis, systemic bioavailability and multi-target therapeutic efficacy through a highly rigorous, multi-disciplinary lens. Greater insight into enzymatic regulation and metabolic engineering will be paramount to enable the scalable production of rare flavonoids with optimized, bioactive scaffolds. Additionally, deciphering the precise influence of chemical structural variations, food-processing matrices and the host gut microbiota on absorption remains crucial. Finally, well-designed human studies and improved delivery strategies are needed to validate clinical relevance and enhance the translational potential of flavonoids.
